# Triplex Real-Time PCR without DNA Extraction for the Monitoring of Meningococcal Disease

**DOI:** 10.3390/diagnostics8030058

**Published:** 2018-08-30

**Authors:** Melissa J. Whaley, Laurel T. Jenkins, Fang Hu, Alexander Chen, Seydou Diarra, Rasmata Ouédraogo-Traoré, Claudio T. Sacchi, Xin Wang

**Affiliations:** 1 Centers for Disease Control and Prevention, Atlanta, GA 30329, USA; mwhaley@cdc.gov (M.J.W.); knt9@cdc.gov (L.T.J.); wwg2@cdc.gov (F.H.); lur5@cdc.gov (A.C.); 2 Institut National de Recherche en Santé Publique, Bamako 00223, Mali; seydsous@yahoo.fr; 3 Centre Hospitalier Universitaire Pédiatrique Charles de Gaulle, Ouagadougou 01, Burkina Faso; ramaouedtra@yahoo.fr; 4 Instituto Adolfo Lutz, São Paulo 01246-902, Brasil; ctsacchi@gmail.com

**Keywords:** meningococcal meningitis, direct multiplex real-time PCR, *Neisseria meningitidis*, meningococcal serogroups

## Abstract

Detection of *Neisseria meningitidis* has become less time- and resource-intensive with a monoplex direct real-time PCR (drt-PCR) to amplify genes from clinical specimens without DNA extraction. To further improve efficiency, we evaluated two triplex drt-PCR assays for the detection of meningococcal serogroups AWX and BCY. The sensitivity and specificity of the triplex assays were assessed using 228 cerebrospinal fluid (CSF) specimens from meningitis patients and compared to the monoplex for six serogroups. The lower limit of detection range for six serogroup-specific drt-PCR assays was 178–5264 CFU/mL by monoplex and 68–2221 CFU/mL by triplex. The triplex and monoplex showed 100% agreement for six serogroups and the triplex assays achieved similar sensitivity and specificity estimates as the monoplex drt-PCR assays. Our triplex method reduces the time and cost of processing CSF specimens by characterizing six serogroups with only two assays, which is particularly important for testing large numbers of specimens for *N. meningitidis* surveillance.

## 1. Introduction

Serotype and serogroup determination are vital in monitoring vaccine impact and guiding vaccine strategies in the prevention of bacterial meningitis [1,2,3]. *Neisseria meningitis* (Nm) is one of the leading causes of bacterial meningitis worldwide [1,4]. Meningococcal serogroups A, B, C, W, X, and Y are commonly associated with invasive disease [1,5]. The distribution of these serogroups varies over time, geographically, and with vaccine implementation [1,3,6]. Laboratory methods are used to identify serogroup and assess the changes in serogroup distribution [1,4,5]. Although culture is considered the gold standard of diagnosis, molecular diagnostic methods, such as real-time polymerase chain reaction (rt-PCR), provide an improved sensitivity in detection and higher throughput, with results available in hours instead of days from specimens with or without viable meningococci [1,4]. Based on the capsule locus genes (NmA-*csaB*, NmB-*csb*, NmC-*csc*, NmW-*csw*, NmX-*csxB*, and NmY-*csy*), traditional singleplex and multiplex rt-PCR that require DNA extraction prior to PCR amplification were developed and validated [1]. The testing process, time, and costs continued to decrease with the introduction of DNA polymerase enzymes resistant to PCR inhibitors, which allowed for the direct detection of meningococcal serogroups from clinical specimens without the need for DNA extraction [4,7,8]. Monoplex direct rt-PCR (drt-PCR) assays were evaluated for all six serogroups and demonstrated similar or better sensitivity when compared to the traditional singleplex rt-PCR assays [4]. In this study, we evaluated two triplex drt-PCR assays for the detection of meningococcal serogroups AWX and BCY, with the aim of further increasing testing efficiency.

## 2. Materials and Methods

### 2.1. Triplex drt-PCR Assays and Lower Limit of Detection

The same optimized primer and probe concentrations, thermal profile (1 cycle of 95 °C for 10 min, and 50 cycles of 95 °C for 15 s and 60 °C for 1 min), mastermix (PerfeCTa qPCR ToughMix from Quanta Biosciences, Gaithersburg, MD, USA), reactions volume (25 µL, 2 µL of which was cerebrospinal fluid, CSF), and Stratagene Mx3005P instruments (Agilent Technology, Santa Clara, CA, USA) were used for monoplex direct and triplex direct rt-PCR assays [4]. While all probes used in the monoplex drt-PCR assays were labeled with FAM fluorophore, probes in each triplex drt-PCR assay were labeled with FAM, HEX, or Quasar670, a CY5 alterative, fluorophore as specified in Table 1 [4]. The fluorophore/serogroup pairs were chosen based on assay performance during the assessment of monoplex serogroup assays and information obtained during the validation of a serogroup triplex assay designed for use with extracted DNA [1].

For all PCR assays, positive specimens were defined as having cycle threshold (*C*_t_) values of 35 or less, while negative specimens had *C*_t_ values of 40 or more [1]. Specimens with *C*_t_ values between 35 and 40 underwent repeat testing with and without a 10-fold dilution; if *C*_t_ values remained greater than 35, then specimen was considered negative.

To determine the lower limit of detection (LLD) of the triplex drt-PCR assays, 10-fold serial dilutions of a bacterial suspension with known concentration, using quality control strains for each serogroup, separately were prepared in Brain Heart Infusion broth for enumeration of colony forming units (CFU) per milliliter (mL), and in pooled cerebrospinal fluid (pooled CSF) for triplicate drt-PCR testing. The CFU/mL was plotted against the mean *C*_t_ for each dilution, and the CFU/mL yielding a *C*_t_ value of 35 was the LLD.

### 2.2. Comparison to Monoplex drt-PCR Assays

The same CSF specimens, from Burkina Faso (*N* = 26, 2010–11), Mali (*N* = 87, 2009–12), Brazil (*N* = 58, 2007–8), United States (*N* = 52, 2001–16), and other countries (*N* = 5, 2010–14), were tested by triplex drt-PCR assays and the corresponding monoplex serogroup assay within the same experiments. These CSF specimens were collected through routine surveillance, underwent confirmatory testing at the corresponding national reference laboratory, and were unlinked from patient identifiers. This project was determined by the Centers for Disease Control and Prevention (CDC) to be non-human subjects research, and Institutional Review Board review was not required.

Since patient care was of the utmost importance, CSF specimens were collected after treatment and therefore, culture was not available for all CSF specimens. Of the 228 CSF specimens, 54 had culture results. The majority were negative by culture and PCR (42/54, 78%), while the 12 positive for *N. meningiditis* by culture were determined to be either serogroups A or W (8 NmA and 4 NmW). Without a consistent reference standard, results from the six validated serogroup-specific drt-PCR assays in the monoplex were used to calculate the percent positive (*N* = 183 CSF) and percent negative (*N* = 45 CSF) agreement of the triplex drt-PCR assays, as estimates of specificity and sensitivity [9].

To assess overall agreement, monoplex and triplex drt-PCR assays were compared pairwise by *C*_t_ values. Summary statistics (mean, standard deviation, minimum, median, and maximum) were calculated for the raw *C*_t_ values by serogroup and assay, and for the *C*_t_ differences (triplex − monoplex) for each specimen. Due to non-normal distribution of the data, a nonparametric sign test was used to assess the significance of the differences between mean *C*_t_ values. The level of agreement between the monoplex and triplex drt-PCR assays were depicted in serogroup-specific Bland-Altman Plots, in which *C*_t_ differences (*C*_t Triplex_ − *C*_t Monoplex_) were plotted against the mean *C*_t_ values of the two assays [(*C*_t Triplex_ + *C*_t Monoplex_)/2] [10]. In the Bland-Altman Plots, a reference line indicates the ideal zero difference; upper and lower limits of agreement were calculated as the mean C_t_ difference +/− 1.96 multiplied by the standard deviation [10]. Kappa analysis was also completed to further assess the overall agreement between the monoplex and triplex drt-PCR assays [11]. For these analyses, SAS 9.3 (Cary, NC, USA) was used.

## 3. Results and Discussion

### 3.1. Characteristics of Meningococcal Serogroup Monoplex and Triplex drt-PCR Assays

The LLD range for the triplex drt-PCR assays was 68–2221 CFU/mL, which is comparable for all respective serogroup-specific monoplex drt-PCR assays tested in parallel with the triplex (178–5264 CFU/mL) (Table 1), which is consistent with the results in the previous monoplex drt-PCR evaluation (168–1606 CFU/mL) [4]. These ranges are well within the typical bacterial load in CSF from a meningitis patient, 10^3^ to 10^5^ CFU/mL [1]. The NmA-*csaB* assay had a higher LLD than other serogroup rt-PCR assays in both traditional multiplex and direct triplex methods, which is consistent with the previous observation [1]. The LLDs for the triplex assays were lower than for the monoplex assays even though the same PCR reaction reagents and concentrations were used in the two assays. The cause of the lower LLDs for the triplex assays is unclear, but it is possible that the higher total concentration of primers and probes within the reaction (due to the presence of three primer/probe sets) contributed to a more ideal reaction efficiency.

This triplex drt-PCR method assesses serogroups in two combinations (AWX and BCY) but could be further optimized to account for geographical differences in serogroup distribution by modifying the serogroup combinations and fluorophores used. However, these modifications would require additional validation. In a region of sub-Saharan Africa with high incidence of meningitis, known as the Meningitis Belt, serogroup A disease has decreased after the introduction of the monovalent serogroup A vaccine and outbreaks have resulted from serogroups C, W, and X at lower frequency and in smaller population sizes than previous serogroup A outbreaks [3,12]. Serogroups B, C, and Y have predominately caused meningococcal disease in countries outside of the Meningitis Belt, although a recent increase in serogroup W disease has been observed [3,13]. When considering different fluorophores, it is important to refer to the dye and instrument compatibility, and to note that the NmA-*csaB* assay previously labeled with CY5 failed to detect serogroup A [1].

As estimates of sensitivity and specificity, triplex drt-PCR results were compared with the monoplex drt-PCR results for the same positive and negative CSF specimens. Based on this comparison, triplex drt-PCR assays maintained 100% positive and negative agreement (Table 1), suggesting similar specificity and sensitivity between the monoplex and triplex assays. Sensitivity and specificity of the triplex drt-PCR assays may be further assessed when a collection of culture-positive CSF specimens come available. 

### 3.2. Pairwise Comparison to Six Monoplex Serogroup drt-PCR Assays

In a pairwise comparison between serogroup-specific monoplex and triplex assays, the difference in *C*_t_ values, for positive CSF tested in parallel, ranged between −3.3 to 2.3 cycles (Table 2). A similar range was observed between duplicates in the traditional singleplex rt-PCR assays (−2.9 to 3.4 cycles), indicating an agreement between the monoplex and triplex drt-PCR assays [1]. Mean *C*_t_ values and standard deviations between the monoplex and triplex assays were very similar. As determined by the sign test, mean *C*_t_ differences were not significant except for serogroup C assays, which had a mean *C*_t_ difference of less than a full cycle (−0.7). This significance was observed previously in the traditional singleplex and multiplex comparison, for serogroup C assays, using CSF from Brazil [1].

**Figure 1:**
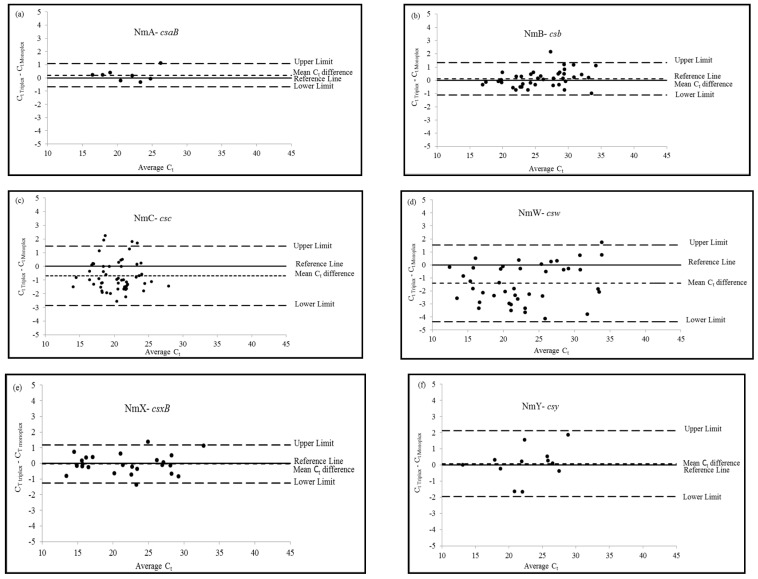
Bland-Altman Plots resulting from the pairwise comparison of *C*_t_ values between triplex and monoplex direct real-time PCR assays by meningococcal serogroups: (**a**) NmA-*csaB*; (**b**) NmB-*csb*; (**c**) NmC-*csc*; (**d**) NmW-*csw*; (**e**) NmX-*csxB;* and (**f**) Nm-*csy*. Level of agreement between the two assays by serogroup (*C*_t Triplex_ − *C*_t Monoplex_), also known as observed mean difference (small dash line), is presented as a function of the average *C*_t_ values of the two assays [(*C*_t Triplex_ + *C*_t Monoplex_)/2]. Reference lines (solid line) indicate the ideal zero difference. Upper and lower limits of agreement (large dash lines) defined by mean *C*_t_ difference +/− 1.96 × SD.

The serogroup-specific Bland-Altman Plots (Figure 1) showed good agreement between the monoplex and triplex drt-PCR assays. For the six serogroup-specific assays, most *C*_t_ differences were within the limits of agreement and close to the zero-difference reference line. Consistent with previous results, a few positive CSF specimens (8 of the 183) were outside of the upper or lower limits. These results indicated that the triplex and monoplex assays were not considerably different. Agreement between triplex and monoplex assays in pairwise comparison by serogroup was further confirmed with kappa analysis. The kappa coefficient was 1 for serogroups A, B, C, W, and Y assays and 0.973 for the serogroup X assay, indicating very good agreement for all assays. Since a limited number of positive CSF were available in this evaluation (*N* = 8 and 12 respectively), additional analysis may be needed to assess the level of agreement for NmA-*csaB* and NmY-*csy* assays with a higher number of specimens.

## 4. Conclusions

In conclusion, the two triplex drt-PCR assays demonstrated similar agreement to that of the monoplex drt-PCR assays for all six serogroups causing invasive disease. By allowing for the detection of six serogroups in two reactions, triplex assays further conserve clinical specimens, and reduce costs and process time. With this increased PCR testing efficiency, laboratories, both domestic and international, may provide meningococcal serogroup information in less time. Serogroup information is essential for local and global disease surveillance, epidemic and outbreak response, and assessing vaccine impact and developments [1,2,3]. Laboratories may also use the triplex assays to resolve ambiguous serogroup results obtained from standard serological procedures [2,5]. Through an expanded validation, the triplex drt-PCR assays may be assessed for the testing of various specimen types, which is increasingly important with the recent clusters of cases with atypical meningococcal disease presentations [13,14,15,16,17].

## Figures and Tables

**Table 1:** Characteristics of direct monoplex and triplex real-time PCR assays for meningococcal serogroup determination.

Assay ^1^	Fw/RV/Pb-Fluorophore ^2^	QC Strains ^3^	Monoplex LLD ^4^ (CFU/mL)	Triplex LLD (CFU/mL)	Triplex PP, PN ^5^
AWX					100% (75/75), 100% (45/45)
NmA-*csaB*	900/600/300 nM-HEX	M7060	5264	2221	
NmW-*csw*	300/100/200 nM-FAM	M7034	197	128	
NmX-*csxB*	600/600/200 nM-Quasar670	M8210	178	68	
BCY					100% (108/108), 100% (45/45)
NmB-*csb*	900/300/200 nM-Quasar670	M5178	420	286	
NmC-*csc*	300/300/300 nM-FAM	M3045	1780	1407	
NmY-*csxB*	900/300/300 nM-HEX	M2578	1232	765	

^1^ Assay named for the included *Neisseria meningitidis* (Nm) serogroup, followed by capsule locus gene; ^2^ Fw, forward primer; RV, reverse primer; Pb, probe; ^3^ QC, quality strains used to determine LLD [5]; ^4^ LLD, lower limits of detection, defined as CFU/mL yielding a *C*_t_ value of 35; ^5^ Positive percent agreement (PP) and negative percent agreement (PN) calculated by using monoplex drt-PCR assays as a reference.

**Table 2:** Summary statistics from a pairwise comparison of *C*_t_ values between triplex (T) and monoplex (M) meningococcal serogroup real-time PCR assays, directly testing on positive cerebrospinal fluid (CSF) specimens.

Assay	N ^1^	*C*_t_ Values				
		Mean (mean ± 1.96 × SD)	SD ^2^	Min ^3^	Med ^4^	Max ^5^
AWX						
NmA-*csaB* (T) ^6^	8	21.4	3.5	16.5	21.3	26.8
NmA-*csaB* (M)	8	21.2	3.4	16.3	21.3	25.7
Difference	8	0.19 (−0.63, 1.01)	0.4	−0.3	0.2	1.1
NmW-*csw* (T)	41	22.7	6.2	12.2	22.1	34.7
NmW-*csw* (M)	41	23.0	6.0	12.5	22.0	32.2
Difference	41	−0.37 (−2.75, 2.00)	1.2	−3.3	−0.23	1.7
NmX-*csxB* (T)	26	21.5	5.7	13.0	21.8	33.3
NmX-*csxB* (M)	26	21.6	5.6	13.8	22.1	32.2
Difference	26	−0.04 (−1.26, 1.19)	0.6	−1.4	−0.1	1.4
BCY						
NmB-*csb* (T)	40	25.8	4.7	16.8	25.7	34.8
NmB-*csb* (M)	40	25.7	4.5	17.2	25.5	34.1
Difference	40	0.1(−1.11, 1.34)	0.6	−1.0	0.1	2.1
NmC-*csc* (T)	56	20.0	2.8	13.3	20.4	27.2
NmC-*csc* (M)	56	20.7	2.8	14.8	21.0	28.6
Difference	56	−0.68 (−2.86, 1.49)	1.1	−2.6	−1.0	2.3
NmY-*csy* (T)	12	22.6	4.7	13.1	22.6	29.8
NmY-*csy* (M)	12	22.6	−4.4	13.1	22.3	27.9
Difference	12	0.08 (−1.97, 2.12)	1.0	−1.7	0.2	1.9

^1^ N, total number of positive CSF tested; ^2^ SD, standard deviation; ^3^ min, minimum; ^4^ med, median; ^5^ max, maximum; ^6^ (T), triplex drt; (M), monoplex drt-PCR.

## Abbreviations

CFU/mL	colony forming units per milliliters
CSF	cerebrospinal fluid
*C*_t_	cycle threshold
drt-PCR	direct real-time polymerase chain reaction
LLD	lower limit of detection
Nm	*Neisseria meningitidis*
rt-PCR	real-time polymerase chain reaction
